# Outcomes of Low-Temperature Stress on Biological Alterations within Pothos (*Epipremnum aureum*) Leaves

**DOI:** 10.3390/life12091432

**Published:** 2022-09-14

**Authors:** Yanqing Wu, Xiang Cai, Yuhan Tang

**Affiliations:** 1 Joint International Research Laboratory of Agriculture and Agri-Product Safety, The Ministry of Education of China, Institutes of Agricultural Science and Technology Development, Yangzhou University, Yangzhou 225009, China; 2 College of Horticulture and Landscape Architecture, Yangzhou University, Yangzhou 225009, China

**Keywords:** *Epipremnum aureum*, low-temperature stress, reactive oxygen species, antioxidant enzyme activity

## Abstract

Pothos (*Epipremnum aureum*) is a commonly used indoor ornamental foliage, particularly in the middle and lower regions of the Yangtze River in China. It typically grows in the tropical area, and it is yet unclear whether prolonged winter temperatures cause plant damage and impact its development. In this study, the *E. aureum* chilling injury response was explored by maintaining it at 1 °C. Based on the acquired results, low-temperature stress (LTS) induced wilting and yellowing of leaves and diminished chloroplast pigment concentrations, particularly the chlorophyll b content. LTS also induced overproduction of reactive oxygen species (ROS) within *E. aureum* and enhanced the relative electrical conductivity and superoxide dismutase activity. In addition, with prolonged LTS, the anatomical structure of *E. aureum* was severely damaged, resulting in a marked reduction in the photochemical activity of the photosystem Ⅱ reaction center and suppressed photosynthesis. Moreover, results of the transcriptomic analysis revealed that LTS induced the expression of genes involved in the α-linolenic acid metabolic pathway, plant hormone network, host plant–pathogen association, and MAPK axis, suggesting that LTS would activate its resistant response to cold stress. These results unraveled the physiological and transcriptomical response of *E. aureum* to chilling injury, which would lay a theoretical foundation for the cultivation of low-temperature-tolerant varieties of *E. aureum*.

## 1. Introduction

Being an important environmental stress factor, cold stress, caused by low-temperature stress (LTS), has an adverse effect on plant growth, development, and yield. Particularly, in recent years, LTS, brought on by an abnormally altered global climate, caused massive losses to the agricultural industry [1]. Plant cold stress is generally divided into two categories: freezing and chilling injuries [2]. Freezing injury is caused by the prolonged plant exposure to low temperatures (i.e., below 0 °C), which may freeze the internal tissues, thus resulting in irreversible damage and death of plants [3,4,5]. Alternately, chilling injury occurs when plants are exposed to low but not freezing temperatures (i.e., above 0 °C) [6]. This typically results in impaired plant growth and development, as well as reduced yield, fruit quality, and ornamental value, but not plant death. For instance, in crops, chilling injury to rice (*Oryza sativa*) seedlings, caused by ‘cold late spring’ weather in March and April, leads to leaf yellowing, slow seedling growth, stunted growth, wilting, and reduced tiller yields, all of which ultimately impact rice yield [7]. In case of fruits, mango (*Mangifera indica*) is a tropical fruit, and chilling injury, caused by improper storage, can negatively impact fruit quality, thereby resulting in lenticel discoloration and circular lesion spread, pitting, off-flavor, and pulp discoloration [8]. In terms of ornamental plants, *Phalaenopsis aphrodite* is known to suffer from chilling injury at a temperature of 2–7 °C. Under such conditions, its mesophyll cells collapse, its leaves become yellow, water gets immersed, and sometimes it develops sunken variegated leaves, which ultimately affects its ornamental values [9].

Pothos (*Epipremnum aureum*) is a perennial evergreen vine belonging to the *Araceae* family. It is native to the Soro Islands and is commonly referred to as Potho, Hunter’s Robe, Devil’s Ivy, or Ivy Arum [10]. Nowadays, *E. aureum* is the most commonly used indoor foliage plant all over China. This is primarily due to its ornamental values as it has luscious green leaves. It also has a purifying function. It serves as a natural ‘air purifier’, which greatly reduces the amount of ozone in the environment, and removes pollutants such as, xylene, formaldehyde, and benzene from indoor air [11]. Being a native of the tropical rain forest area in the Soro Islands, *E. aureum* prefers a warm and humid climate, with an optimal growth temperature of 20–32 °C, and it is susceptible to chilling injury or freezing damage in a low-temperature environment. Currently, *E. aureum* is widely used for its ornamental and purification functions in the middle and lower regions of the Yangtze River. Since the winter temperature in these regions is around 0 °C, it is easy to cause chilling injury to *E. aureum*. Therefore, it is critical to explore effective methods of improving its cold tolerance in the future.

Most studies on *E. aureum* are focused on its purifying functions [12,13]; however, few investigated its responses to stress, such as chilling injury. To elucidate the physiological and transcriptomical response of *E. aureum* to chilling injury, chloroplast pigment contents, reactive oxygen species (ROS), water content, relative electrical conductivity, antioxidant enzymatic activities, chlorophyll fluorescence variables, anatomical observation, and transcriptomic analysis were assessed in this study. This investigation will enhance the understanding of the *E. aureum* physiological and transcriptomical response to chilling injury, which would lay a theoretical foundation for the cultivation of low-temperature-tolerant varieties of *E. aureum.*

## 2. Materials and Methods

### 2.1. Plant Materials and Treatments

*E. aureum* plants that exhibited strong and steady development were purchased from the Huaduhui horticultural market in Yangzhou. *E. aureum* plants grown in soil were separated into two groups of 15 pots each. One group was maintained at 1 °C and was designated as the LTS group. The other group was maintained at 20 °C and was designated as the control group. Both groups received an air humidity of 60%, with light intensity of 30,000 lx for 14 h in the daytime and 0 lx for 10 h at nighttime. Leaf specimen and other data were collected on the 0, 7, 14, 21, and 28-day post-treatment, and three *E. aureum* plants were arbitrarily chosen as replicates for individual treatments. Following chlorophyll fluorescence parameters and ROS measurements, the collected leaves were sliced into smaller portions prior to fixation in 2.5% glutaraldehyde and ultrastructural observation. The remaining leaves were instantly frozen in liquid nitrogen prior to storage in −80 °C for further analyses.

### 2.2. Chloroplast Pigment Contents Measurement

Chloroplast pigment content measurements were performed as described by Lewis et al. [14]. In short, 0.2 g fresh weight (FW) leaves were weighed prior to the introduction of 12 mL of 95% ethanol and subsequent grinding of the leaves until they completely turned white. This was followed by filtration via a filter paper into a 25 mL volumetric flask. To ensure maximum yield, both the filter paper and residue were washed multiple times. Next, ethanol was introduced to stabilize the volume (V) and then mixed well. The chloroplast pigment extract absorbance values (A_665_ and A_649_ representing wavelengths 665 and 649 nm, respectively) were next recorded, and chlorophyll a and b in the samples were computed based on the following equations: chlorophyll a content = (13.95 × A_665_ − 6.88 × A_649_) × V/FW; chlorophyll b content = (24.96 × A_649_ − 7.32 × A_665_) × V/FW. To elucidate the total chlorophyll content, chlorophyll a and b contents were added together.

### 2.3. ROS Determination

Hydrogen peroxide (H_2_O_2_) accumulation was assessed via diaminobenzidine (DAB) staining [15], which was prepared via the introduction of a 0.1 mg·mL^−1^ DAB staining solution (pH 5.0) to a 50 mM Tris-acetate buffer. Fresh leaves were soaked in the prepared dye without light for 24 h, prior to boiling in 95% alcohol for 15 min, removal, and photography.

The superoxide radicals (O_2_^−^) accumulation was assessed via a kit (Shanghai Haring Biotechnology Co. Ltd., Shanghai, China). In short, fresh leaves were rapidly sliced using a double-sided blade while leaving the major veins intact. Next, the sliced samples were rinsed in distilled water on a slide prior to the addition of 10 μL dihydroethidium (DHE) fluorescent dye, incubation without light at 37 °C for 20 min, followed by observation and photography under a fluorescence microscope (Axio Imager D2, ZEISS, Dusseldorf, Germany).

### 2.4. Leaf Water Content Measurement

The leaf water content was determined based on the Fang et al. report [16]. In short, an analytical balance (Suzhou Scientific Instruments Co., Ltd., Suzhou, China) was used to record the weights of fresh leaves as FW. Next, leaves were incubated in a 105 °C oven (9423A, Shanghai Jinghong Experimental Equipment Co., Ltd., Shanghai, China) for 5 min, prior to drying in a 65 °C oven to constant weight, which was then recorded as the dry weight (DW). Leaf water content (%) = (FW − DW)/FW × 100%.

### 2.5. Relative Electrical Conductivity Measurement

The relative electrical conductivity determination followed a protocol described by Xu et al. [17]. In short, fresh leaves were rinsed in ultrapure water prior to punching approximately 1 cm diameter holes to acquire small leaf disks. About 0.1 g leaves were then weighed and inserted into a syringe with ultrapure water. Following vacuuming, the leaves and 20 mL deionized water were placed in a glass tube, which was then maintained at room temperature (RT) for 4 h. The mixture was then mixed well before the initial solution conductivity (C1) measurement via a conductivity meter (DDS–307-A, Ray Magnetic Instrument Co., Ltd., Shanghai, China). The glass tube was heated in a boiling water bath for 30 min prior to the conductivity (C2) measurement. The REC (%) was computed as follows: C1/C2 × 100%.

### 2.6. Antioxidant Enzymatic Activities Determination

The enzymatic activities of superoxide dismutase (SOD), peroxidase (POD), and catalase (CAT) were determined using the corresponding kits (Suzhou Keming Biotechnology Co., Ltd., Suzhou, China) following the kit directions.

### 2.7. Chlorophyll Fluorescence Parameters Measurement

Chlorophyll fluorescence parameters were next measured using a chlorophyll fluorescence spectrometer (Heinz Walz GmbH, Nuremberg, Germany) after the plants were incubated without light for over 2 h. PAM Win software was employed for the measurement and computation of the PSII photochemical maximum quantum yield (variable fluorescence (*Fv*)/maximum fluorescence (*Fm*), PSII photochemistry (Y(II), nonphotochemical quenching coefficient (*qN*), and quantum yield of nonregulated energy dissipation (Y(NO)).

### 2.8. Anatomical Observation

The anatomical observation referenced the Zhao et al. [18] study. Briefly, fresh leaves were sliced into smaller portions of 1 × 1 cm prior to fixation in 2.5% glutaraldehyde at 4 °C for a minimum of 4 h. The leaves were then rinsed thrice in a 0.1 M phosphate buffer for 15 min each prior to a secondary fixation in 1% osmium tetroxide for 4 h. Subsequently, they were dehydrated in 100% acetone and acetone-containing anhydrous sodium sulfate for 15 min each. This was followed by embedding in Spurr resin, sectioning, and double-staining with uranyl acetate and lead citrate. Lastly, the mesophyll cells and chloroplasts were visualized and photographed under a transmission electron microscope (TEM) (HT7700, HITACHI, Tokyo, Japan).

### 2.9. RNA Isolation, cDNA Library Generation, and Sequencing

Two groups of samples (control and 1 °C LTS at day 28), with three replicates, were employed for RNA-seq. Total RNA was isolated with a mirVana™ miRNA ISOlation Kit (Ambio-1561, North Augusta, SC, USA) following the kit directions, with subsequent RNA quantification via an Agilent 2100 Bioanalyzer (Agilent Technologies, Santa Clara, CA, USA). Samples with an RNA integrity number ≥7.0 and 28S/18S ratio ≥0.7 were used for subsequent analyses. cDNA libraries were generated via a TruSeq Stranded mRNA LTSample Prep Kit (Illumina, San Diego, CA, USA) per the kit directions. Lastly, these libraries were sequenced on an Illumina HiSeq 4000 platform to obtain 150 bp paired-end reads.

### 2.10. Sequence Assembly, Annotation, Identification, and Enrichment Analysis

Raw data (raw reads) processing employed Trimmomatic [19]. Reads with ploy-N and those with low quality were eliminated to achieve clean reads, which were then entered into the expressed sequence tag clusters (contigs) and into a transcript via Trinity [20] (version: 2.4) using the paired-end technique. The largest mRNA, based on the sequence similarity and length, was selected as a unigene for further analyses. Subsequently, unigene function was identified using unigene annotation based on the NCBI nonredundant (NR), SwissProt, and Clusters of orthologous groups for eukaryotic complete genome (KOG) databases via Blastx [21] and a cutoff E-value of 10^−5^. Next, proteins that received the largest hits to the unigenes were used to assign the corresponding functional roles. Using SwissProt, gene ontology (GO) stratification was conducted via mapping of the relationship between the SwissProt and GO term, followed by mapping of the unigenes to the Kyoto Encyclopedia of Genes and Genomes (KEGG) [22] database to screen for possible metabolic networks. The FPKM [23] and read count values of individual unigenes were computed via bowtie2 [24] and eXpress [25]. Differentially expressed genes (DEGs) were recognized via the DESeq [26] functions estimate size factors and nbinom test, and *p* value < 0.05 and fold change > 2 were set as the significance thresholds. The DEG-based GO and KEGG network enrichment analyses were carried out via R depending on the hypergeometric distribution. Lastly, TFs were recognized via the analysis of the InterProScan domain patterns in sequences with elevated coverage.

### 2.11. qRT-PCR Validation

A BIO-RAD CFX Connect™ Optics Module (Bio-Rad, Hercules, CA, USA) was employed for the qRT-PCR-based analysis of gene transcription. The same samples that underwent RNA-seq were employed in the qRT-PCR assessment. In short, isolated leaf RNA (1 μg) was converted to cDNA using the superscript first-strand synthesis system (PrimeScript^®^ RT Reagent Kit With gDNA Eraser, TaKaRa, Osaka, Japan). *EaActin* (DN 31460) was employed as an endogenous control. All targeted primers were synthesized by Beijing Qingke Biotechnology Co., Ltd., and are summarized in Table 1. The qRT-PCR (TaKaRa, Osaka, Japan) reaction was conducted with SYBR^®^ Premix Ex Taq™ (Perfect Real Time) using the following parameters: 55 °C for 2 min, an initial denaturation step at 95 °C for 30 s,  40 cycles at 95 °C for  5 s, 55 °C for 15 s, and 72 °C for 30 s. The relative gene expression was computed using the 2^−ΔΔCt^ comparative threshold cycle (Ct) formula [27].

### 2.12. Statistical Analysis

All experiments were replicated three times and then averaged. SAS/STAT (version 6.12, SAS Institute, Chicago, IL, USA) was utilized for the analysis of variance, and graph plotting was conducted in GraphPad 8.0 software.

## 3. Results

### 3.1. Influence of LTS on Chloroplast Pigment Concentrations

**Figure 1:**
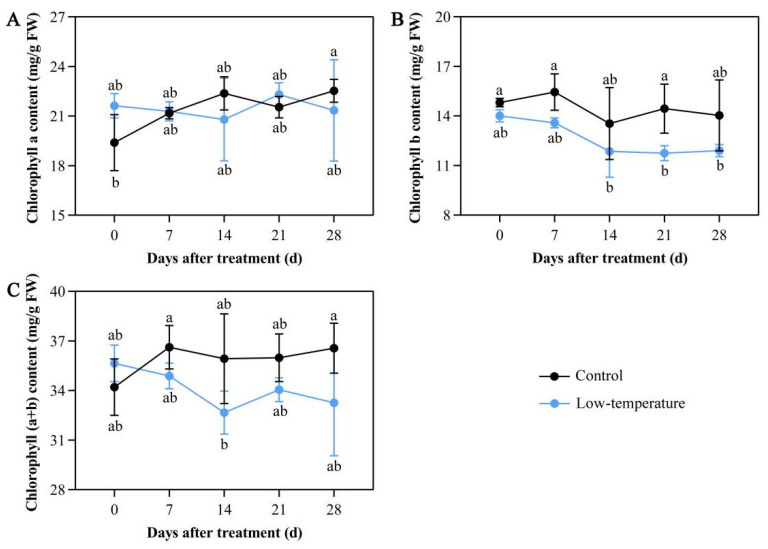
Low-temperature stress (LTS) impacts chloroplast pigment contents in *E. aureum* leaves. (**A**) Chlorophyll a levels; (**B**) chlorophyll b levels; (**C**) chlorophyll (a + b) levels. Values denote mean ± standard deviation (SD), and different letters represent significantly lower or higher values compared with controls, as evidenced by Duncan’s multiple range test (*p* < 0.05).

Under continuous LTS, *E. aureum* growth was severely altered. Relative to the controls, *E. aureum* under LTS gradually turned yellow and lost their luster, and some even turned black and wilted. Following 7 days of LTS, the chlorophyll b and (a + b) contents were drastically diminished compared with the controls (Figure 1A,C). Moreover, with continued LTS, the chlorophyll b and (a + b) contents were consistently lower than those of the controls (Figure 1A,C). However, LTS did not alter the chlorophyll a levels (Figure 1B). This may be due to the reduced chlorophyll content owing to diminished photosynthesis caused by LTS, which resulted in the gradual yellowing and wilting of the leaves.

### 3.2. Influence of LTS on ROS Concentrations

**Figure 2:**
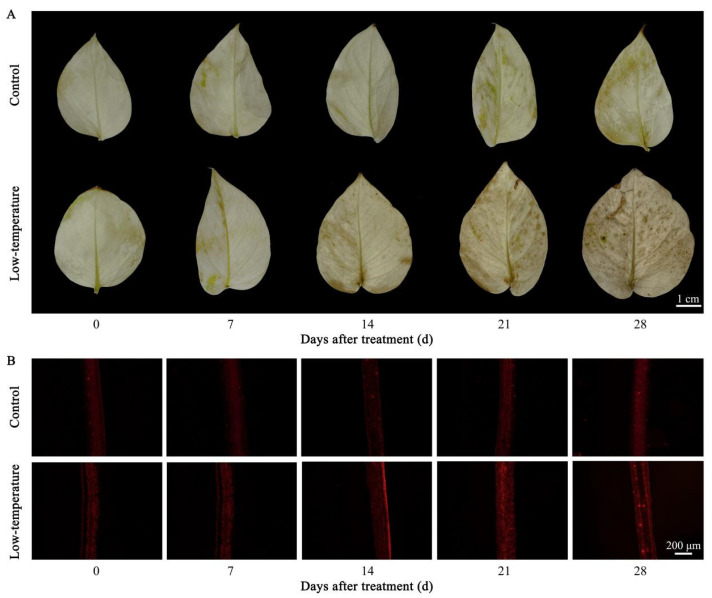
Low-temperature stress (LTS) influences reactive oxygen species (ROS) concentrations in *E. aureum* leaves. (**A**) Hydrogen peroxide (H_2_O_2_) accumulation, as evidenced by diaminobenzidine (DAB) staining; (**B**) superoxide radicals (O_2_^·−^) accumulation, as evidenced by fluorescence prob.

LTS causes oxidative damage in plants, which leads to the excessive production of ROS. H_2_O_2_ and O_2_^·−^ are essential regulators of ROS accumulation. To measure the H_2_O_2_ production, DAB staining was used, and the leaf color represented the membrane lipid peroxidation status. A darker staining represented a higher level of membrane lipid peroxidation or membranal damage within the leaves. Following 7 days of LTS, the H_2_O_2_ accumulation status in the leaves was not markedly different from that in the controls. However, after 14 days of LTS, there was considerably more H_2_O_2_ accumulation in the leaves compared with the controls. Moreover, after 28 days of LTS, the degree of H_2_O_2_ accumulation in the leaves reached the maximum (Figure 2A). To measure O_2_^·−^ accumulation, we employed DHE fluorescent probes, and a strong red fluorescence represented enhanced O_2_^·−^ levels in the mesophyll cells. As illustrated in Figure 2B, following 7 days of LTS, the status of O_2_^·−^ accumulation within the leaves was not markedly different from that within the controls. However, following 14 days of LTS, the status of O_2_^·−^ accumulation within the leaves was elevated compared with the controls and remained consistently higher than that within the controls. These findings suggested that LTS markedly enhanced the ROS accumulation in the *E. aureum* leaves, causing severe oxidative damage to the plants.

### 3.3. Influence of LTS on Leaf Water Content

**Figure 3:**
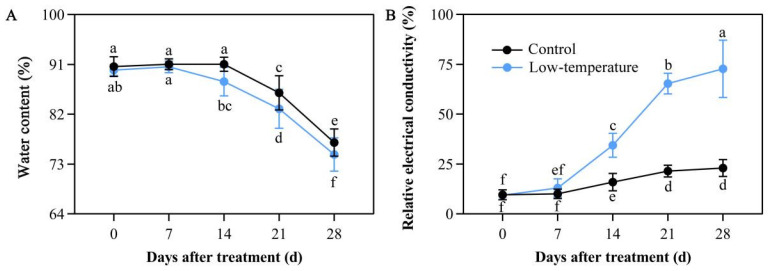
Low-temperature stress (LTS) impacts water content (**A**) and relative electrical conductivity (**B**) in *E. aureum* leaves. Values denote mean ± standard deviation (SD), and different letters represent markedly diminished or elevated values, relative to controls, based on Duncan’s multiple range assessment (*p* < 0.05).

Cold stress exposure causes marked imbalance in water metabolism. Relative to the controls, LTS markedly reduced the water content in the leaves. A 7-day LTS did not alter the leaf water content compared with the controls. However, a 14-day LTS drastically diminished the leaf water content compared with the controls. Moreover, under continuous LTS, the leaf water content was substantially lower than the controls (Figure 3A). These findings indicated that LTS disrupts the delicate balance of water metabolism within *E. aureum*.

### 3.4. Influence of LTS on Relative Electrical Conductivity

Relative electrical conductivity can represent the stress-induced damage status of plants. Following 7 days of LTS, the relative leaf conductivity started to rise compared with the controls. In addition, with continuous LTS, the relative leaf conductivity rose to a maximum value at 28 days of LTS. The increase, compared with the controls, was by 3.16 folds (Figure 3B), indicating that the plant damage status gradually enhanced with increasing days of LTS exposure.

### 3.5. Influence of LTS on Antioxidant Enzymatic Activities

**Figure 4:**
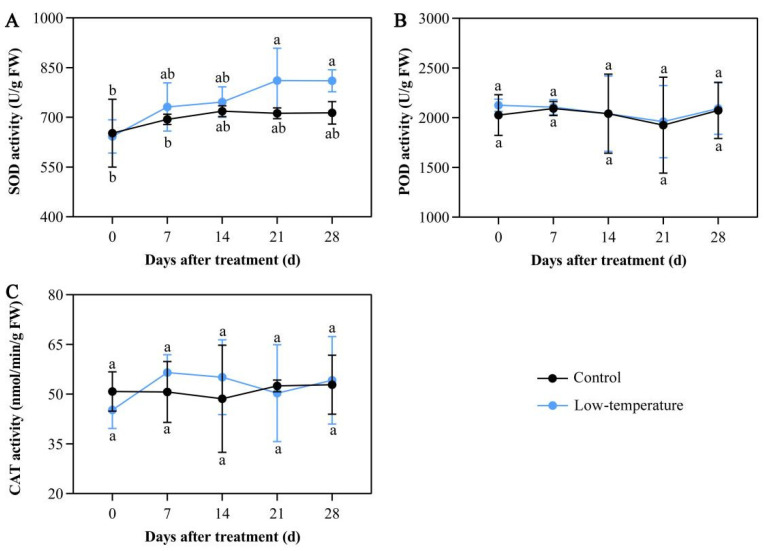
Low-temperature stress (LTS) disrupts antioxidant enzymatic activities in *E. aureum* leaves. (**A**) Superoxide dismutase (SOD) activity; (**B**) peroxidase (POD) activity; (**C**) catalase (CAT) activity. Values denote mean ± standard deviation (SD), and different letters represent markedly reduced or elevated values, relative to controls, based on Duncan’s multiple range assessment (*p* < 0.05).

Antioxidative enzyme systems are known to scavenge excess ROS under abiotic stress. Following LTS, the activities of major antioxidant enzymes such as SOD, POD, and CAT were not substantially altered (Figure 4A–C). The SOD activity slightly increased, but did not reach significance (Figure 4A). These results indicated that *E. aureum* failed to scavenge ROS by inducing peroxidase activity under LTS.

### 3.6. Influence of LTS on Chlorophyll Fluorescence Parameters

**Figure 5:**
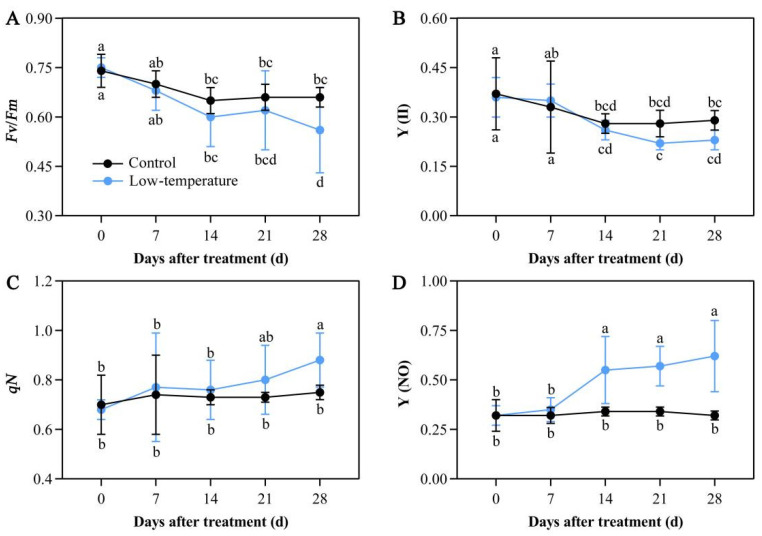
Low-temperature stress (LTS) impacts chlorophyll fluorescence parameters in *E. aureum* leaves. (**A**) Variable fluorescence (*Fv*)/maximum fluorescence (*Fm*) value; (**B**) PSII photochemistry (Y(II)) value; (**C**) nonphotochemical quenching coefficient (*qN*) value; (**D**) quantum yield of nonregulated energy dissipation (Y(NO)) value. Values denote mean ± standard deviation (SD), and different letters represent markedly decreased or increased values, relative to controls, based on Duncan’s multiple range assessment (*p* < 0.05).

LTS strongly regulated chlorophyll fluorescence parameters. Under LTS, *Fv*/*Fm* of dark-adapted leaves and Y(II) values significantly decreased relative to the controls. Following 14 days of LTS, the *Fv*/*Fm* value started to reduce relative to the controls. Following 28 days of LTS, *Fv/Fm* reached the lowest value and was drastically diminished by 16.22%, as opposed to the controls. Following 21 days of LTS, the Y(II) value also started to reduce relative to the controls (Figure 5A,B). Meanwhile, the *qN* and Y(NO) values were markedly increased in the LTS leaves. Following 14 days of LTS, the Y(NO) value started to rise relative to the controls. Following 21 days of LTS, the *qN* value started to rise compared with the controls. Additionally, by the 28th day of LTS, both the *qN* and Y(NO) values reached their peaks, with increases of 17.94% and 95.01% of control leaves, respectively (Figure 5C,D). These results suggested that LTS severely disrupted the electron transport and photochemical activity of the PSII photosystem in plants, thus affecting plant photosynthesis.

### 3.7. Influence of LTS on Leaf Ultrastructure

**Figure 6:**
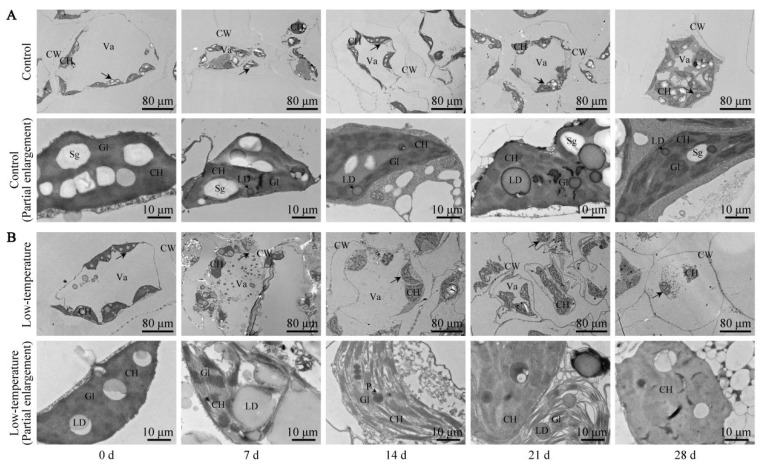
Low-temperature stress (LTS) impacts mesophyll cells and chloroplasts in *E. aureum* leaves. (**A**) Control group; (**B**) LTS group. Lower image is an enlarged version of a portion, indicated by black arrow, of upper image. CH: chloroplast; CW: cell wall; Va: central large vacuole; Sg: starch granule; LD: lipid droplet; P: plastid pellet; Gl: basal lamellae.

The *E. Aureum* mesophyll cell ultrastructure was observed via a TEM. As illustrated in Figure 6, the control leaf ultrastructure was complete with a large central vacuole, and the peripheral cytoplasm contained elliptical chloroplasts. The chloroplast envelope was complete, the internal grana were compact and of comparable size, and the grana lamellae were neatly arranged. There was a small amount of lipid droplets, and one or more starch granules were distributed within the chloroplast. Following LTS, the mesophyll cell ultrastructure significantly changed. Under continuous LTS, the mesophyll cells became gradually deformed, and the separation of the cytoplasm and cell wall was more prominent. On the 21st day of LTS, the mesophyll cells became severely deformed and irregular, and on the 28th day of LTS, the large central vacuole was completely ruptured, and the number of starch granules in the chloroplast was drastically reduced. These findings suggested that LTS completely destroyed the integrity of the chloroplast structure, which eventually affected plant photosynthesis.

### 3.8. Mapping and Quantitative Evaluation of Illumina Sequences

Six cDNA libraries, namely Control_1, Control_2, Control_3, Treatment_1, Treatment_2, and Treatment_3, were generated from the total RNA. All RNA-seq data associated with this investigation were uploaded to the NCBI Sequence Read Archive (SRA) database under the accession number PRJNA851036. Overall, 40.98 G clean data were acquired, with the effective data volume distribution of individual samples in 6.55 G–7.25 G; the Q30 base distribution was over 94.32–94.83%; and the average GC content was 51.54% (Table 2). Subsequently, 66,695 unigene strips were spliced with total and mean lengths of 67,423,826 bp and 1010.93 bp, respectively. The database annotations of unigenes were as follows: 28,413 (42.60%) genes to the NR library; 20,059 (30.08%) genes to the Swissprot library; 5839 (8.75%) genes to the KEGG library; 15,842 (23.75%) genes to the KOG library; 25,290 (37.92%) genes the to eggNOG library; 17,295 (25.93%) genes to the GO library; and 17,087 (25.62%) genes to the Pfam library (Table 3). The reads to the unigene had an alignment rate between 83.26% and 84.99%.

### 3.9. DEGs Identification and Enrichment Analyses

**Figure 7:**
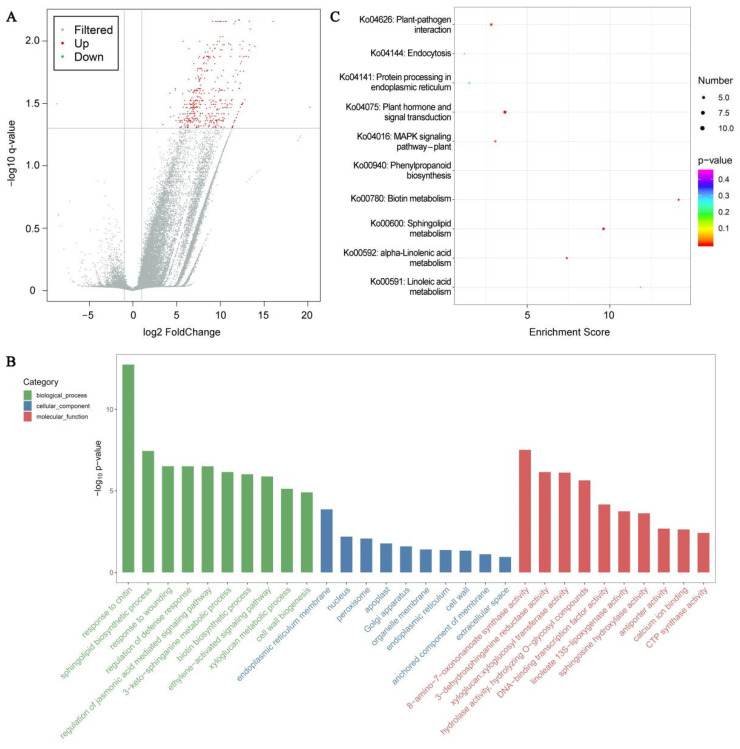
Low-temperature stress (LTS) impacts transcriptomic analysis in *E. aureum* leaves. (**A**) Plot of differentially expressed genes (DEGs) in control vs. LTS leaves; (**B**) Leading 30 GO term distributions of DEGs under LTS; (**C**) Leading 10 KEGG pathway enrichments under LTS.

Unigene expression was computed via FPKM, and the results of the correlation analysis of the unigene expression between samples revealed a correlation coefficient distribution in the range 0.9982~0.9993. Based on *Q* value < 0.05 and |log2 (fold change)| >1, 439 DEGs were identified, with 438 upregulated and 1 downregulated expressions (Figure 7A). Based on *Q* value < 0.05 and DEGs number ≥ 2, 30 GO terms with 10 leading GO term enrichments in all categories were selected (Figure 7B). In the biological process (BP) category, marked DEG enrichments were in the chitin response (GO:0010200, 14.04, 15; GO term ID, enrichment score, DEGs number), sphingolipid biosynthetic process (GO:0030148, 15.75, 8), and wounding response (GO:0009611, 7.37, 11). The endoplasmic reticulum membrane (GO:0005789, 2.79, 17), nucleus (GO:0005634, 1.32, 65), and peroxisome (GO:0005777, 3.42, 6) were the three leading items in the cellular component (CC). In the molecular function (MF) category, the 8-amino-7-oxononanoate synthase activity (GO:0008710, 47.25, 5), 3-dehydrosphinganine reductase activity (GO:0047560, 27.80, 5), and xyloglucan:xyloglucosyl transferase activity (GO:0016762, 18.29, 6) were the most notably enriched. Furthermore, to elucidate the roles of DEGs, they were mapped to the reference specification path within the KEGG database. Based on *Q* value < 0.05 and DEGs number ≥ 2, DEGs were significantly enriched in seven KEGG pathways (Figure 7C). Among them, the plant hormone network (ko04075, 3.79, 12; KEGG pathway ID, enrichment score, DEGs number), alpha-linolenic acid metabolism (ko00592, 7.48, 5), linoleic acid metabolism (ko00591, 11.90, 3), plant–pathogen association (ko04626, 2.93, 8), and MAPK axis–plant (ko04016, 3.16, 6) were strongly related to cold tolerance.

**Figure 8:**
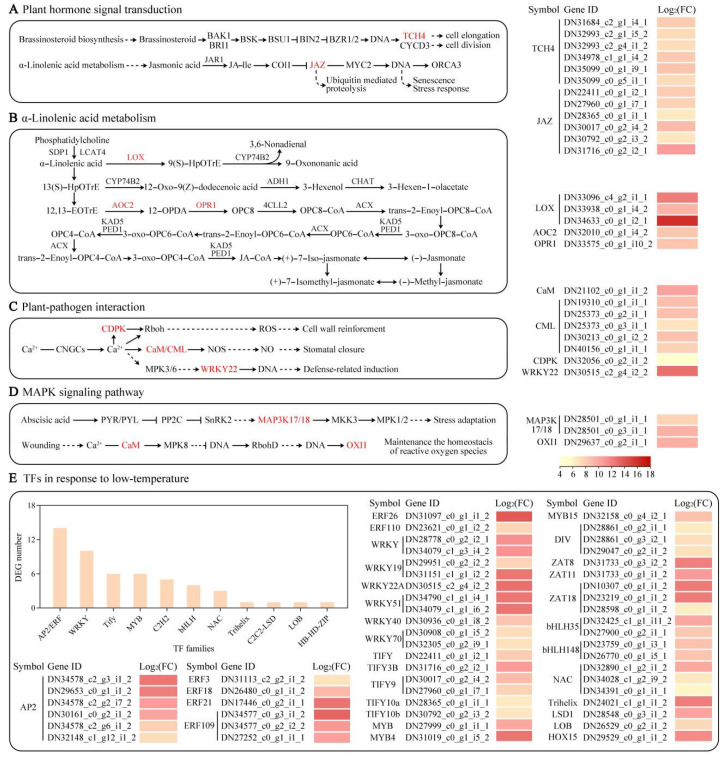
The heat maps of DEGs relevant to plant hormone signaling pathway (**A**), alpha-linolenic acid metabolism (**B**), plant–pathogen association (**C**), MAPK axis–plant (**D**), and TFs (**E**) under low-temperature stress (LTS).

To better identify the core DEGs related to cold tolerance, metabolism pathway and heat map analyses were performed. In the plant hormone signal transduction, the xyloglucan:xyloglucosyl transferase (*TCH4*; DN31684_c2_g1_i4_1, DN32993_c2_g1_i5_2, DN32993_c2_g4_i1_2, DN34978_c1_g1_i4_2, DN35099_c0_g1_i9_1, and DN35099_c0_g5_i1_1) and jasmonate ZIM domain-containing protein (*JAZ*; DN22411_c0_g1_i2_1, DN27960_c0_g1_i7_1, DN28365_c0_g1_i1_1, DN30017_c0_g2_i4_2, DN30792_c0_g2_i3_2, and DN31716_c0_g2_i2_1) were associated with the brassinosteroid (BR) and jasmonic acid (JA) networks, respectively, and were markedly elevated under LTS (Figure 8A). The alpha-linolenic acid metabolism was related to the JA signaling pathway, and LTS induced the expression of key genes such as lipoxygenase (*LOX*; DN33096_c4_g2_i1_1, DN33938_c0_g1_i4_2, and DN34633_c0_g1_i2_1); allene oxide cyclase (*AOC2*; DN32010_c0_g1_i4_2); and 12-oxophytodienoate acid reductase (*OPR1*; DN33575_c0_g1_i10_2) (Figure 8B). In the plant–pathogen association and MAPK axis–plant, calmodulin (*CaM*; DN21102_c0_g1_i1_2); calmodulin-like protein (*CML*; DN19310_c0_g1_i1_1, DN25373_c0_g2_i1_1, DN25373_c0_g3_i1_1, DN30213_c0_g1_i2_2, and DN40156_c0_g1_i1_1); calcium-dependent protein kinase (*CDPK*; DN32056_c0_g2_i1_2); *WRKY22* (DN30515_c2_g4_i2_2); serine/threonine-protein kinase (*OXI1*; DN29637_c0_g2_i1_1); and mitogen-activated protein kinase kinase kinase 17/18 (MAP3K17/18; DN28501_c0_g1_i1_1 and DN28501_c0_g3_i1_1) were also markedly enhanced under LTS (Figure 8C,D). In addition, based on the RNA-seq results, 52 DEGs were identified as transcription factors (TF) and were classified into 11 TF families (Figure 8E). Among the recognized TF families with DEG number ≥ 2, AP2/ERF and WRKY were the two top abundant categories, followed by Tify, MYB, C2H2, bHLH, and NAC.

### 3.10. Verification of DEG Expression Profile via qRT-PCR

**Figure 9:**
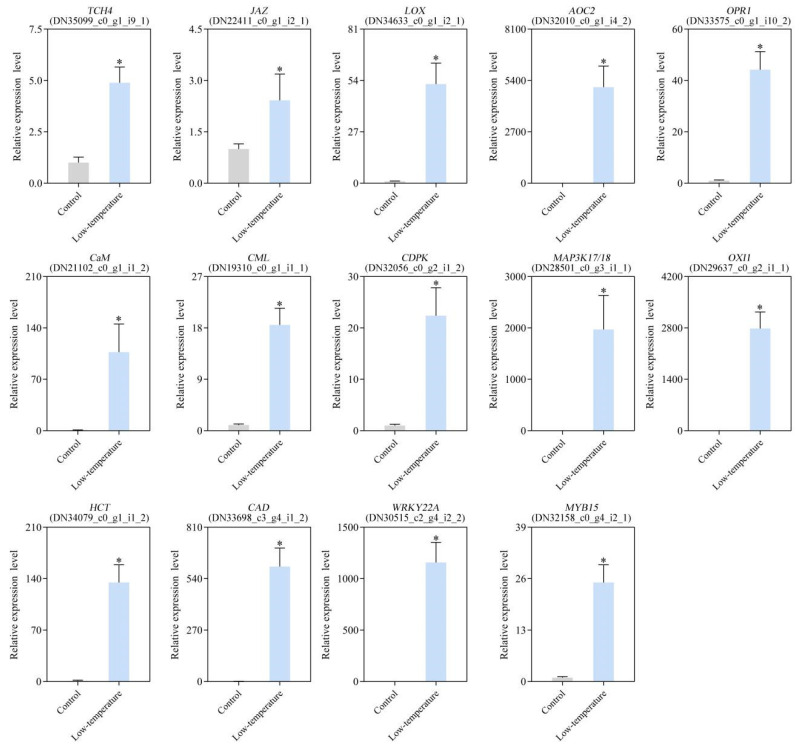
Validation of gene expression profiles under low-temperature stress (LTS) using qRT-PCR. qRT-PCR analysis of 14 specific DEGs. Data are presented as mean ± standard deviation (SD), and ‘*’ indicated significantly lower or higher values, compared with control (*p* < 0.05).

To verify the RNA-seq results, qRT-PCR was carried out. Fourteen DEGs, namely *TCH4* (DN35099_c0_g1_i9_1), *JAZ* (DN22411_c0_g1_i2_1), *LOX* (DN34633_c0_g1_i2_1), *AOC* (DN32010_c0_g1_i4_2), *OPR* (DN33575_c0_g1_i10_2), *CaM* (DN21102_c0_g1_i1_2), *CML* (DN19310_c0_g1_i1_1), *CDPK* (DN32056_c0_g2_i1_2), *OXI1* (DN29637_c0_g2_i1_1), *MAP3K17/18* (DN28501_c0_g3_i1_1), hydroxycinnamoyl CoA: shikimate hydroxycinnamoyl transferase (*HCT*; DN34079_c0_g1_i1_2), cinnamyl alcohol dehydrogenase (*CAD*; DN33698_c3_g4_i1_2), *WRKY22* (DN30515_c2_g4_i2_2), and *MYB15* (DN32158_c0_g4_i2_1), were evaluated. *EaActin* (DN31460_c0_g1_i2_1) was used as a reference gene for qRT-PCR. The expression profiles of the 14 qRT-PCR-validated DEGs were highly consistent with the transcriptome sequencing results, suggesting great reliability of the DEG analysis data (Figure 9).

## 4. Discussion

Chilling injury refers to plants in a temperature environment lower than the lowest limit of their optimal growth temperature [28], which inhibits their growth to a certain extent. LTS affects various physiological activities of plants, which is manifested by reduced growth, loss of water, withering of tissues and organs, yellow leaves with water stains, and even death [29]. In this study, the *E. aureum* leaves turned yellow and tarnished, and even turned black and withered under LTS. The leaf water content gradually decreased from day 0 to day 28 after LTS relative to the controls. This may be due to a reduction in the ability of the root system to absorb and transport water under the influence of cold stress, as well as the stomata closure that results in plant water loss. The leaf water content represents the water retention ability of leaves to a certain extent. When plants are subjected to cold stress, water metabolism is disrupted. This was previously demonstrated in rice [30]. Taken together, these results indicated that the cold stress degree of *E. aureum* can be partially reflected by the water content of leaves.

LTS affects plant photosynthesis and induces ROS overproduction, which is reflected by the accumulation of H_2_O_2_ and O_2_^·−^ [31,32]. Generally, ROS accumulation leads to lipid peroxidation and plant cell death, which eventually suppresses plant development [33]. Herein, H_2_O_2_ and O_2_^·−^ accumulations were enhanced with sustained LTS. This is consistent with studies on bananas (*Musa nana*) [34] and wheat (*Triticum aestivum*) [35]. LTS usually leads to membrane permeability and electrolyte leakage, which, in turn, results in the extravasation of cytosol and an increase in relative electrical conductivity [36]. Moreover, the relative *E. aureum* electrical conductivity also gradually augments under LTS, which is in accordance with the Fu et al. report [37]. This further verifies that LTS promotes lipid membrane peroxidation and enhances membrane permeability, thereby damaging the cellular membrane.

Antioxidative enzyme systems, comprising of SOD, POD, and CAT, sequester excess ROS under abiotic stress [38]. Under LTS, an enhanced antioxidant enzymatic activity represents a stronger ability to resist environmental stress [39]. In plant cells, SOD reduces the superoxide to H_2_O_2_, which is rapidly decomposed into O_2_ and H_2_O by CAT and POD [40,41]. Herein, we demonstrated that LTS slightly enhanced the SOD activity, similar to its activities in potato (*Solanum tuberosum*) [42] and *Calendula officinalis* [43]. This suggested that *E. aureum* can, to a certain extent, clear LTS-induced ROS by promoting the antioxidant enzymatic activity in order to reduce the cell membrane damage, and better adapt to the external environment. However, in coconuts (*Cocos nucifera*) [44], the POD and CAT activities demonstrated a gradual rising trend with decreasing temperature.

LTS accelerates the PSII electron transport by diminishing the PSII photochemical activity [45]. PSII is an integral part of the photosynthetic mechanism, and it serves an essential function in light energy conversion and electron transport. *Fv*/*Fm* strongly represents the photochemical efficiency of the PSII reaction, and it is frequently employed as a chlorophyll fluorescence kinetic parameter for the representation of photosynthetic mechanism, such as photochemical activity [46]. Chlorophyll fluorescence dissipation includes *qP* and *qN*; the latter protects the photosynthetic apparatus from intense light damage. With increasing *qN*, plants are able to tolerate excess light energy, owing to the better protection of the photosynthetic system. Y(NO) reflects the light damage severity, and, therefore, an increasing Y(NO) value represents further accumulation of light energy within the plant [47]. In maize (*Zea mays*), Zhang et al. [48] reported that the PSII reaction center is destroyed following LTS, and the plant accumulates excess light energy, which further reduces the photochemical efficiency manifested by a reduction in *Fv*/*Fm* and Y(II) and subsequent elevation in Y(NO). In this study, *Fv*/*Fm* and Y(II) were diminished with sustained LTS, whereas the *qN* and Y(NO) values enhanced, corroborating the results in *Cycas panzhihuaensis* [49]. This indicated that the PSII damage in *E. aureum* under LTS continued to increase with the extension of treatment days, and the plant activated the photoprotective mechanism to reduce the LTS-based damage.

The chloroplast ultrastructure is known to alter under LTS [50]. Herein, LTS caused massive alterations to the mesophyll cell ultrastructure of *E. aureum*. The inner vesicle was inflated, and the grana lamellae were irregularly arranged. These phenomena were consistent with the results in tung (*Vernicia fordii*) [51]. Together, this indicated that LTS destroyed the chloroplast structural integrity, thereby inducing irreversible damage to the plant photosynthetic system, with eventual photosynthesis suppression.

To protect themselves against cold, plants often alter their morphological, physiological, biochemical, and molecular characteristics [52]. Herein, RNA-seq was employed to screen for LTS-associated genes. Following LTS, 99.71% DEGs were upregulated compared with the controls. The results of the GO enrichment analysis demonstrated that LTS induced the modulation of the jasmonic acid network (GO:2000022), defense response (GO:0031347), calcium ion interaction (GO:0005509), and so on. In addition, using KEGG enrichment analysis, enrichments were seen in genes that participated in the plant hormone network (ko04075), alpha-linolenic acid metabolism (ko00592), plant–pathogen association (ko04626), and MAPK axis–plant (ko04016) under LTS, which was similar to the data from wucai (*Brassica campestris* L.) [53], tea (*Camellia sinensis*) [54], and pepper (*Capsicum annuum* L.) [55] under cold stress. These networks are modulated by various sensing and signaling genes associated with JA, Ca^2+^ signal network, and MAPK cascade, and this study identified additional genes that were involved in the signal sensing and signaling networks activated by LTS.

Signaling networks are critical for the response to LTS [56]. JA and its derivatives are known to regulate abiotic stress resistance [57] via interaction with JAZ subgroup members [58]. Multiple reports suggested that JA modulates cold tolerance via the activation of the CBF gene expression [59]. JA-Ile induces JAZs binding to the F-box protein COI1, which initiates the ubiquitin-based degradation of JAZs, thereby enabling ICE1/2 association and *CBF* activation [60]. Herein, six *JAZ* genes were elevated under LTS, which affected the JA signaling pathway, thereby improving cold tolerance. Moreover, Ca^2+^ influx is another critical-membrane-based alteration that occurs under LTS [61]. The transient elevation of the [Ca^2+^]_cyt_ content via the Ca^2+^ influx is detected by an array of Ca^2+^ sensing proteins such as CaM, which undergo structural alteration and activation. Ca^2+^-loaded CaM/CML associations modulate a myriad of downstream proteins, which directly or indirectly regulate plant responses to environmental stressors such as LTS [62]. Herein, LTS-associated Ca^2+^ sensors were identified. Under LTS, one *CaM*, five *CML*, and one *CDPK* genes were markedly elevated suggesting that these Ca^2+^ signaling-associated genes promote enhanced cold tolerance. Additionally, the ROS-induced MAPK axis regulates cold stress signaling in *Arabidopsis* [63]. The MAPK axis involves MAP kinase kinase kinase (MAP3K or MEKK), MAP kinase kinase (MAP2K, or MKK), and MAP kinase (MAPK, or MPK) [64], and, in this study, two *MAP3K17/18* were elevated following LTS. Furthermore, TFs play an essential role in modulating the gene expression in plants under abiotic stress [65,66]. In *Arabidopsis*, glutathione (GSH) modulates the *MPK3* levels via *WRKY40* in response to LTS [67]. *Panax ginseng MYB4* overexpression in *Arabidopsis* enhances seedling tolerance to drought, salt, and cold conditions [68]. Trifoliate orange (*Poncirus trifoliata* (L.) Raf.) *ERF109* overexpression confers augmented cold tolerance in transgenic tobacco and lemon plants, whereas inhibition of *PtrERF109* in trifoliate orange based on virus-induced gene silencing (VIGS) leads to enhanced cold susceptibility [69]. Herein, LTS-responsive TFs were screened, namely TFs such as *WRKY40*, *MYB4*, and *ERF109*, which likely serve essential functions in cold tolerance modulation within *E. aureum.* Given these pieces of evidence, future investigations can elucidate the specific roles of these select TFs in metabolic networks to better comprehend their modulatory role in *E. aureum*.

## 5. Conclusions

In conclusion, this investigation examined alterations in various physiological indices and transcriptome within *E. aureum* following LTS to explore its tolerance to chilling injury. Under LTS, *E. aureum* leaves turned yellow and lost their moisture and luster. This was accompanied by excess ROS production, decreased *Fv*/*Fm* and Y(II) values, as well as severely impaired chloroplast structural integrity, which eventually led to oxidative damage and the negative modulation of photosynthesis. With sustained LTS, SOD activity was slightly increased, and the *qN* and Y(NO) values were elevated, compared with the controls. Therefore, *E. aureum,* to a certain extent, reduced oxidative damage and mobilized the photoprotective mechanism to enhance its LTS tolerance. LTS also induced the expression of genes associated with the JA, Ca^2+^ signal network, and MAPK axis such as *JAZ*, *CaM*, *CML*, and *MAP3K17/18* genes. In addition, it enhanced the expression of TFs, namely *WRKY40*, *MYB4,* and *ERF109,* in response to chilling injury in an attempt to enhance cold tolerance in *E. aureum*. These results unraveled the physiological and transcriptomical response of *E. aureum* to chilling injury, which would lay a theoretical foundation for the cultivation of low-temperature-tolerant varieties of *E. aureum.*

## Figures and Tables

**Table 1:** Primer sequences used in qRT-PCR reactions in this study.

Gene Symbol	Gene ID	Primer Pairs	Tm (°C)	Product Size (bp)
*Actin*	DN31460_c0_g1_i2_1	F:AACTGCTCCTGAAAATCG R:CAACATCAATGACAACACCT	48.8	152
*TCH4*	DN35099_c0_g1_i9_1	F:CGTCACCGCCTACTACCTA R:TTGCTGTTCCCTATTCCC	53.8	139
*JAZ*	DN22411_c0_g1_i2_1	F:GAGTTGTCTCCAGGATAAA R:GATTAGAGGGCTGAAGAG	52.6	126
*LOX*	DN34633_c0_g1_i2_1	F:GCTCAACTTCGGGCAGTA R:AGTGCGTGGACAGCGTAT	56.8	197
*AOC2*	DN32010_c0_g1_i4_2	F:GACAGGTGAGGGGAAGGTA R:GGAATGTAACTCCAAGGAAA	54.1	191
*OPR1*	DN33575_c0_g1_i10_2	F:GGAGAAACACGGAAGGAG R:CAACCATACACCCCAAAA	52.4	159
*CAM*	DN21102_c0_g1_i1_2	F:ATTCAGGAGGATGGCAGA R:CGAAAAGGCAATTCAAGTC	53.9	193
*CML*	DN19310_c0_g1_i1_1	F:CACAAGCATCCACATCAG R:GCACCACCACGACTACTA	50.8	115
*CDPK*	DN32056_c0_g2_i1_2	F:CGATGTTTTCCCTGTCTA R:CTTGGATGTTATGGGTCTAT	46.6	106
*MAP3K17/18*	DN28501_c0_g3_i1_1	F:ATCAGACATCACCACCGA R:CTGGTATTCCGTTCATCG	53.0	123
*OXI1*	DN29637_c0_g2_i1_1	F:TCTGATGCTTGTTGATTTCG R:AGTCGGAGTTTCCTTGCC	54.3	179
*HCT*	DN34079_c0_g1_i1_2	F:GTGATAAGAGCACCCGAGA R:GCAACGCCACAATAGAGC	53.9	105
*CAD*	DN33698_c3_g4_i1_2	F:TGTCAGTGCCATTGTAGG R:GATGTCACCAGGTTCTCG	53.9	136
*WRKY22A*	DN30515_c2_g4_i2_2	F:AGACTCCCAAGTCCAAAA R:GCTGCATCTGTAATAACCC	52.0	155
*MYB15*	DN32158_c0_g4_i2_1	F:GCTGGTCTGTTGAGGTGT R:GTTCTTGATTTCGTTGTCTG	52.4	189

**Table 2:** Quality metrics of unigenes in *E. aureum* leaves with control and low-temperature treatment (LTS).

Sample	Raw Reads	Raw Bases	Clean Reads	Clean Bases	Valid Bases	Q30	GC
Control_1	50.04 M	7.51 G	48.97 M	6.88 G	91.70%	94.73%	51.52%
Control_2	48.37 M	7.26 G	47.37 M	6.64 G	91.48%	94.83%	51.15%
Control_3	48.46 M	7.27 G	47.43 M	6.69 G	91.99%	94.77%	51.46%
Low-temperature_1	47.10 M	7.06 G	45.89 M	6.55 G	92.70%	94.34%	51.75%
Low-temperature_2	51.73 M	7.76 G	50.55 M	7.25 G	93.38%	94.47%	51.85%
Low-temperature_3	50.08 M	7.51 G	48.79 M	6.97 G	92.81%	94.32%	51.48%

**Table 3:** Annotations of unigenes in *E. aureum* leaves with control and low-temperature treatment (LTS).

Database	NR	Swissprot	KEGG	KOG	eggNOG	GO	Pfam
Annotated number	28,413	20,059	5839	15,842	25,290	17,295	17,087
Percentage (%)	42.6	30.08	8.75	23.75	37.92	25.93	25.62

## Data Availability

Not applicable.

## Abbreviations

*AOC2*	allene oxide cyclase
BP	biological process
BR	brassinosteroid
*CAD*	cinnamyl alcohol dehydrogenase
*CaM*	calmodulin
CAT	catalase
*CDPK*	calcium-dependent protein kinase
CH	chloroplast
*CML*	calmodulin-like protein
CW	cell wall
DAB	diaminobenzidine
DEGs	differentially expressed genes
DHE	dihydroethidium
DW	dry weight
*Fm*	maximum fluorescence
*Fv*	variable fluorescence
FW	fresh weight
Gl	basal lamellae
GO	gene ontology
*HCT*	hydroxycinnamoyl CoA: shikimate hydroxycinnamoyl transferase
JA	jasmonic acid
*JAZ*	jasmonate ZIM domain-containing protein
KEGG	Kyoto Encyclopedia of Genes and Genomes
H_2_O_2_	hydrogen peroxide
KOG	clusters of orthologous groups for eukaryotic complete genomes
LD	lipid droplet
*LOX*	lipoxygenase
LTS	low-temperature stress
*MAP3K17/18*	mitogen-activated protein kinase kinase kinase 17/18
NR	nonredundant
O_2_^·−^	superoxide radicals
*OPR1*	12-oxophytodienoate acid reductase
*OXI1*	serine/threonine-protein kinase
P	plastid pellet
POD	peroxidase
*qN*	nonphotochemical quenching coefficient
ROS	reactive oxygen species
RT	room temperature
SD	standard deviation
Sg	starch granule
SOD	superoxide dismutase
SRA	sequence read archive
*TCH4*	xyloglucan:xyloglucosyl transferase
TEM	transmission electron microscope
TF	transcription factors
V	volume
Va	central large vacuole
VIGS	virus-induced gene silencing
Y(II)	PSII photochemistry
Y(NO)	quantum yield of nonregulated energy dissipation
